# Chronic Inflammatory Demyelinating Polyradiculoneuropathy (CIDP) Developing During Tacrolimus Treatment: A Case Series

**DOI:** 10.1002/mus.70025

**Published:** 2025-09-17

**Authors:** Matthew C. Evans, Roberto Bellanti, Samer Dahdaleh, Kushan Karunaratne, Simon Rinaldi, Jane Pritchard, Stuart Viegas

**Affiliations:** ^1^ Imperial College Healthcare NHS Trust London UK; ^2^ Nuffield Department of Clinical Neurosciences University of Oxford Oxford UK

**Keywords:** calcineurin inhibitor, CIDP, immunosuppression, neurotoxicology, tacrolimus

## Abstract

**Introduction/Aims:**

Chronic Inflammatory Demyelinating Polyradiculoneuropathy (CIDP) can occasionally emerge during treatment with tacrolimus, a commonly used immunosuppressant for solid organ transplantation, and this association is poorly recognized. We describe the clinical presentation, investigations, and disease course of a series of patients who developed CIDP during tacrolimus treatment.

**Methods:**

This is a retrospective case series of six patients with electrophysiologically confirmed CIDP (=2021 EFNS/PNS criteria) during tacrolimus use for solid organ transplantation, evaluated at two UK National Health Service (NHS) trusts between 2017 and 2023. We describe the clinical characteristics, laboratory investigations, neurophysiological features, treatment response, and association with tacrolimus treatment.

**Results:**

CIDP was diagnosed between 5 months and 13 years after initiation of tacrolimus, post cardiac (2), renal (2), lung (1), and combined kidney‐pancreas (1) transplantation. All patients met diagnostic criteria for CIDP. 5/6 patients improved clinically following intravenous immunoglobulin (IVIg). 4/6 patients were switched to either sirolimus or azathioprine without evidence of active disease despite no further treatment. 2/6 patients continued tacrolimus, and both required ongoing IVIg treatment.

**Discussion:**

CIDP may be associated with tacrolimus use in organ transplantation and can occur after years of treatment. IVIg was usually effective in our cohort, and in those who switched to alternative immunosuppression, there was no evidence of active disease after initial treatment.

**Abbreviations**
CIDP: chronic inflammatory demyelinating polyradiculoneuropathy
CMV: cytomegalovirus
EBV: Epstein–Barr virus
EFNS: European Federation Neuroscience Society
EMG: electromyogram
IL‐2: interleukin‐2
IVIg: intravenous immunoglobulins
MAG: myelin‐associateda glycoprotein
NCS: nerve conduction studies
PNS: Peripheral Nerve Society
TNF: tumor necrosis factor

## 1 Introduction

Chronic inflammatory demyelinating polyradiculoneuropathy (CIDP) has a presumed immune‐mediated pathology, with differing lines of evidence variously implicating antibodies, complement, T cells, and macrophages. However, specific antibodies are rarely identified in routine clinical practice, save for those found in autoimmune nodopathies, which are now considered a separate disease entity [1]. Interferon‐y, anti‐tumor necrosis factor (TNF) therapies, and immune checkpoint inhibitors [2, 3] have been reported as causing CIDP. Tacrolimus, a calcineurin inhibitor, disrupts T cell signal transduction and interferes with interleukin‐2 (IL‐2) signaling [4]. This mechanism has been causally implicated in CIDP.

We present a case series of six patients who developed CIDP during tacrolimus therapy for solid organ transplantation in two neuroscience centers in the South of England, comparing their clinical presentation, response to therapies, and relationship to tacrolimus treatment.

## 2 Methods

All patients gave informed consent for their pseudonymized data to be included in this case series. The Imperial College Research Ethics Committee determined that this study did not require ethical review. This is a two‐center (Imperial College Healthcare NHS Trust; Oxford University Hospitals Foundation Trust) chart review and case series for patients diagnosed with CIDP in the context of tacrolimus use for solid organ transplantation. All patients under the care of neuromuscular consultants at these institutions between 2014 and 2022 were identified. We extracted pseudonymised information from electronic patient records, including blood work and neurophysiology data. Nerve conduction studies (NCS) were performed by either a consultant neurophysiologist or a neurophysiology scientist and reported by a consultant neurophysiologist. Skin temperature was maintained between 32°C and 33°C. Standard protocols for inflammatory neuropathy included assessment of sural, superficial peroneal, median, and ulnar sensory nerves, as well as tibial, common peroneal, median, and ulnar motor nerves unilaterally or bilaterally, with some patients additionally having assessment of the radial nerve. For the purposes of this study, we re‐assessed all neurophysiology studies against American Association of Neuromuscular and Electrodiagnostic Medicine (AANEM) normative values [5]. Original diagnoses of CIDP were made according to the previous 2010 European Federation of Neurological Societies/Peripheral Nerve Society (EFNS/PNS) guidelines [6]. We additionally re‐evaluated patients against the most recent 2021 EFNS/PNS guidelines [7].

## 3 Results

Six patients were identified who developed CIDP while on treatment with tacrolimus. Detailed clinical information can be found in Table 1. Patient vignettes are provided in Data S1. Patients ranged from 27 to 68 years of age at the time of diagnosis of CIDP, which occurred between 5 months and 13 years after exposure to tacrolimus. Two patients had heart transplants, two had kidney transplants, one had a lung transplant, and one had a simultaneous kidney‐pancreas transplant. One patient had sensorimotor symptoms/signs limited to the legs, two patients had both upper and lower limb involvement (but face spared) and three patients had symptoms/signs involving all four limbs and the face. Five patients had a subacute course prior to diagnosis and treatment, ranging between 4 and 8 weeks of symptom progression. Patient 5 had a slower course with 15 months of symptoms before diagnosis. One patient (Patient 2) developed respiratory failure requiring mechanical ventilation, and no patient developed clinically significant dysautonomia. In terms of comorbidities that could be confounding in the development of neuropathy, one patient had steroid‐induced diabetes, and another patient had type 2 diabetes. Additionally, three patients had cytomegalovirus (CMV) viraemia.

**TABLE 1:** Summary of clinical findings from the six patients with tacrolimus‐induced CIDP.

Patient (age [years], sex)	Daily tac. dose (duration)	Transplant	Comorbities	Presentation	Other investigations	2021 EFNS/PNS CIDP criteria	IVIg response?	Tacrolimus switched?	Long‐term remission?
1 (27, M)	18 mg (12 years)	Cardiac	Kawasaki Haem. anemia DVT	4 weeks progressive paraesthesia UL and LL distal weakness	MRI brain/spine: normal CSF protein 610 mg/dL Normal CSF cells	Met (motor only)	No	Yes—sirolimus	Yes
2 (51, F)	13 mg (5 months)	Renal	Mesangio‐capillary GN CMV viraemia	6 weeks progressive non‐length‐dependent symptoms Ascending 4‐limb and facial weakness Respiratory failure	CSF protein 144 mg/dL Acellular CSF Ganglioside and paranodal Ab negative	Met (sensory and motor)	Yes	Yes—mycophenolate and prednisolone	Yes
3 (69, M)	4 mg (14 months)	Cardiac	Dilated cardiomyopathy Steroid‐induced diabetes	4 weeks progressive limb distal numbness, progression proximally with UL, LL and facial weakness	CSF protein raised Acellular CSF Ganglioside and paranodal Ab negative	Met (sensory and motor)	Yes	Yes—sirolimus	Yes
4 (60, M)	2 mg (3 years)	Lung	COPD R hemicolectomy RIJ thrombosis CKD CMV viraemia	Numbness hands and feet, progressing over 8 weeks with development of UL and LL weakness	MRI brain/spine: normal CSF acellular, normal protein	Met (sensory and motor)	Yes	No	No Ongoing IVIg
5 (59, F)	2.5 mg (13 years)	Renal	BK viraemia HTN Papillary thyroid Ca	Numbness in feet, progression over 15 months, then prox > distal LL weakness	MRI brain/spine: normal CSF protein 332 mg/L, acellular MAG Ab negative	Met (sensory and motor)	Yes	No	No Ongoing IVIg
6 (55, M)	2 mg (2 years)	Kidney/pancreas	Type 2 diabetes CMV viraemia	8 weeks progressive weakness and paraesthesia LL and UL	MRI brain/spine: normal IgG lambda paraprotein CSF protein 34 mg/dL	Met (sensory and motor)	Yes	Yes—sirolimus	Yes

Abbreviations: CKD, chronic kidney disease; CMV, cytomegalovirus; COPD, chronic obstructive pulmonary disease; CSF, cerebrospinal fluid; EFNS, European Federation of Neurological Sciences; GN, glomerulonephritis; IF, immunofixation; IVIg, intravenous immunoglobulin; Ix, investigations; LL, lower limb; MAG, myelin‐associated glycoprotein; MRI, magnetic resonance imaging; PNS, Peripheral Nerve Society; RIJ, right internal jugular; SPE, serum protein electrophoresis; Tac, tacrolimus; Tx, treatment; UL, upper limb.

With respect to diagnostic testing, four patients had raised cerebrospinal fluid (CSF) protein, with values ranging from 34 to 610 mg/dL. Magnetic resonance imaging (MRI) of the brain and spine was normal in all patients. Ganglioside antibodies were assayed in three patients, paranodal antibodies in two, and myelin‐associated glycoprotein (MAG) in one; all were negative. NCS met 2021 European Academy of Neurology (EAN)/PNS criteria for CIDP in all six patients [7]. Patient 1 met the motor NCS criteria only, with the remaining patients meeting both sensory and motor criteria.

All six patients received intravenous immunoglobulin (IVIg) at 2 g/kg. Only Patient 1 did not show clinically meaningful improvement following IVIg. Four patients were switched from tacrolimus to either sirolimus or azathioprine, and subsequently required no further immunosuppression to maintain remission. Both patients who were maintained on tacrolimus required ongoing IVIg maintenance treatment.

## 4 Discussion

Calcineurin inhibitors form the basis of immunosuppression for solid organ transplantation. Tacrolimus is generally the preferred calcineurin inhibitor owing to its superiority over cyclosporin for the prevention of rejection for a number of organ transplant types [8, 9]. We describe six patients who developed chronic demyelinating neuropathy during tacrolimus use following solid organ transplantation. This association has previously been described in several case reports and small case series (of 2–3 patients) [10, 11, 12, 13, 14, 15, 16, 17, 18, 19, 20, 21, 22], further details of which are summarized in Table [Link], [Link]. Of the previous 19 reported patients with (radiculo)neuropathies complicating tacrolimus use, only 10 demonstrated progression or relapses beyond 28 days [10, 12, 13, 15, 17, 21]. Nine met the updated 2021 EAN/PNS electrophysiological criteria for CIDP. In contrast, 6/9 acutely presenting or indeterminate neuropathies were axonal, so there appears to be a bimodal pattern of neuropathies associated with tacrolimus: an early axonal pathology and later emergence of a chronic, CIDP‐like process with typical demyelinating neurophysiology.

In our series, CIDP occurred in the context of a wide range of tacrolimus doses (2–18 mg daily), and all patients demonstrated therapeutic plasma concentrations for tacrolimus. Furthermore, CIDP emerged and was first diagnosed after months to years of treatment, consistent with previous reports [12]. Regarding treatment response, 5/6 patients had an objective response to IVIg, and patients who were able to switch to alternative immunosuppression maintained remission, while those who remained on tacrolimus required maintenance IVIg, largely in keeping with previous literature, in which sustained remission was achieved by stopping tacrolimus [12, 14, 17, 19], or disease improvement occurred with a dose reduction [15, 16, 18]. However, not all of the previously published studies demonstrate this relationship between improvement in neuropathy and reduction in tacrolimus dose [22]. A review of broader tacrolimus‐induced neurotoxicity including central as well as peripheral manifestations also concluded that stopping or switching tacrolimus therapy usually resulted in remission of the associated complication/toxicity [23].

There are some important caveats of the patients discussed here. First, while most had clear demyelinating features on electrophysiology, meeting the neurophysiological criteria for CIDP, Patient 5 had neurophysiology at presentation more consistent with an axonal neuropathy with reduced/absent motor and sensory amplitudes and evidence of active denervation on EMG. However, follow‐up studies demonstrated the emergence of demyelinating features which would meet the criteria for CIDP diagnosis, suggesting that early studies likely reflected a severe disease phenotype as opposed to primary axonal damage. The same patient also had albuminocytological dissociation, further in keeping with an inflammatory polyradiculoneuropathy.

An additional difficulty in attributing causation to tacrolimus in this population is that often patients with immunosuppression for solid organ transplantation have coexisting viraemia, particularly involving EBV and CMV, and both of these infectious agents have also been associated with acute inflammatory radiculoneuropathies [24, 25]. In our series, three patients had CMV viraemia around the time of development of the neuropathy, although those that improved did so without specific antiviral treatment. Because these immunogenic viruses are largely associated with acute inflammatory (monophasic) polyradiculoneuropathies, it is possible they played a role in the development of neuropathy here.

It is not clear whether there is a direct causal link between CIDP and tacrolimus. However, there is mounting evidence of an association, which may be a class effect, as similar neuropathies have been noted with cyclosporine [22]. Tacrolimus is a calcineurin inhibitor that interferes with T cell function (via disrupted IL‐2 signalling) [4], so it is mechanistically possible that it could interact with known disease mechanisms in CIDP [26, 27, 28].

In summary, this series highlights a potential association between CIDP and tacrolimus treatment across a range of solid organ transplant recipients, occurring at various doses and even after many years of well‐tolerated treatment. While most patients demonstrated a sustained clinical response to IVIg, remission in our cohort was only achieved in those who transitioned to sirolimus or, for one patient, azathioprine. Awareness of this disease entity is important for neurologists and transplant physicians caring for patients with solid organ transplantation. Given the rarity of this potential complication, further large‐scale cohort studies are needed to establish the link between tacrolimus use and demyelinating neuropathy [29], to identify potential risk factors, and to better assess treatment responses.

## Author Contributions

**Matthew C. Evans:** writing – original draft, writing – review and editing, conceptualization, data curation. **Roberto Bellanti:** writing – original draft, writing – review and editing, data curation. **Samer Dahdaleh:** conceptualization, writing – original draft, data curation. **Kushan Karunaratne:** conceptualization, writing – original draft, data curation. **Simon Rinaldi:** supervision, data curation, writing – review and editing. **Jane Pritchard:** data curation, supervision, writing – review and editing. **Stuart Viegas:** supervision, data curation, writing – review and editing.

## Ethics Statement

We confirm that we have read the Journal's position on issues involved in ethical publication and affirm that this report is consistent with those guidelines.

## Consent

We thank all patients for providing informed consent to discuss de‐identified details of their diagnosis and treatment in this manuscript.

## Conflicts of Interest

Simon Rinaldi has received honoraria for lectures given at the request of Excemed, Fresenius, CSL Behring, UCB, argenx, the Beijing Association of Holistic and Integrated Medicine, and the Irish Institute of Clinical Neuroscience. He has been a paid consultant for Argenx, Annexon, Dianthus, Takeda, and Hansa Biopharma. He is an unpaid member of the medical advisory board of the Guillain–Barré syndrome and Related Inflammatory Neuropathies (GAIN) charity and of the Inflammatory Neuropathy Consortium (INC) board. He has previously received reduced registration fees, travel grants, and scientific prizes from the Peripheral Nerve Society. He runs a not‐for‐profit diagnostic testing service for nodal/paranodal antibodies. Stuart Viegas has received an honorarium and travel grants from UCB. The other authors declare no conflicts of interest.

## Supporting information


**Data S1:** Supporting Information.

**Table S1:** Supporting Information.

## Data Availability

The data that support the findings of this study are available on request from the corresponding author. The data are not publicly available due to privacy or ethical restrictions.
