# Supplementary material for: Chronic Inflammatory Demyelinating Polyradiculoneuropathy (CIDP) Developing During Tacrolimus Treatment: A Case Series
Source: Muscle Nerve. 2025 Sep 17;72(6):1289–93. doi: 10.1002/mus.70025 (PMC12599595; doi:10.1002/mus.70025)
Supplement: Supplementary file 1 — Data S1: Supporting Information. [file MUS-72-1289-s002.docx]

**Supplementary materials**

**Patient vignettes**

Patient 1

A 15-year-old boy required a cardiac transplant due to Kawasaki disease. After 12 years of treatment with tacrolimus (18 mg daily), and prednisolone 8 mg daily, he presented with a four-week history of paraesthesia and difficulty walking. He had distal weakness of all four limbs and reduced pinprick sensation below the wrists and knees. Lower limb reflexes were absent whilst those in the upper limbs were supressed. MRI brain and spine with contrast were normal. Neurophysiology was in keeping with a demyelinating polyneuropathy with reduced motor nerve conduction velocities, absent F-waves and conduction block affecting the common peroneal nerve. Cerebrospinal fluid (CSF) examination was acellular with markedly elevated protein (610 mg/dL), matched oligoclonal bands and negative CSF viral polymerase chain reaction (PCR) and cytology. Ganglioside antibodies were negative. He was treated with intravenous immunoglobulin (IVIg) at 2 g/Kg over 5 days with no treatment response. Clinical deterioration continued beyond 8 weeks, at which point tacrolimus was changed to sirolimus and he was started on a reducing course of prednisolone (starting at 60 mg once a day). Two months later, there was significant improvement in the weakness and sensory disturbance. The prednisolone dose was gradually weaned over a few months to 7 mg a day maintenance dose. Nerve conduction studies six months later were normal. He has remained in clinical remission since, seven years later on 7 mg prednisolone and sirolimus for post-transplant immunosuppression.

Patient 2

A 51-year-old female underwent a live-donor renal transplant, after being diagnosed with mesangiocapillary glomerulonephritis 14 years prior. She was dialysis dependent for 5 years, initially with peritoneal followed by haemodialysis. Following transplantation, she was treated with mycophenolate mofetil (750 mg twice daily) and tacrolimus (6.5 mg twice daily). Four months after transplantation, she developed CMV viraemia and was treated with valganciclovir, initially with no neurological symptoms. One month later, she began to develop sensory symptoms in her distal limbs and face, followed, after a few days, by progressive distal and proximal lower limb weakness and mild radicular pain. CSF was acellular with albuminocytological dissociation (protein 144 mg/dL) and negative viral PCR. Neurophysiology was in keeping with an inflammatory demyelinating polyradiculoneuropathy. She received 3 cycles of plasma exchange which provided temporary improvements, but continued to worsen after a few days, with emergence of respiratory failure. The neuropathy progressed beyond 6 weeks from onset. At nadir, the patient had severe quadriplegia, no facial movements, and required mechanical ventilation. Tacrolimus was then stopped, and she was given pulsed intravenous methylprednisolone followed by IVIg and an oral prednisolone wean. Two weeks later, power had improved to MRC grade 3-4/5, artificial ventilation could be discontinued, and she was discharged after another two weeks. On review in clinic six weeks later, power was full and there was no residual sensory deficit. To date, her neuropathy has not recurred.

Patient 3

A 68-year-old male received a heart transplant for dilated cardiomyopathy and was treated with mycophenolate, prednisolone and tacrolimus 4 mg. 14 months later, he developed numbness and tingling in his toes and fingers, which spread proximally over the next month to the knees and wrists. He then lost the ability to open bottles or dress independently and could barely stand unsupported. At first assessment, he had symmetric proximal and distal weakness in all four limbs, with mild bilateral lower motor neurone facial weakness and areflexia. Lumbar puncture demonstrated albuminocytological dissociation, and neurophysiology showed patchy demyelinating changes meeting criteria for CIDP. He was treated with 5 days of IVIg with significant clinical improvement and was discharged for outpatient rehabilitation. He represented 1 month later with deteriorating motor and sensory symptoms, at which point the tacrolimus was exchanged for sirolimus. Over the next 10 days, his weakness, sensory disturbance, and imbalance again improved. On review at two months, he had only residual paraesthesia in the toe and fingertips and remained well without further relapse one year later.

Patient 4

A 57-year-old man had a bilateral lung transplant for severe chronic obstructive pulmonary disease (COPD), which was complicated by caecal perforation requiring a right hemicolectomy. Other past medical history included chronic kidney disease with recurrent CMV viraemia and previous steroid-induced diabetes. His immunosuppression regime was tacrolimus 1 mg twice daily and prednisolone 5 mg daily. Three years after transplantation, he developed enterovirus meningitis followed three weeks later by distal sensory and motor impairment in upper and lower limbs, which gradually progressed over 8 weeks. CSF examination was unremarkable (acellular, normal protein) and MRI of brain and spine were normal. Electrophysiology demonstrated slow conduction velocities, conduction block, prolonged distal motor latencies and absent F waves. He was treated with IVIg (2 g/kg), with clear treatment response within the first week and maximal benefit after 4 weeks. An attempt to switch to sirolimus was made, but this was not tolerated due to side effects, so he continued with tacrolimus treatment after discussion with his transplant team due to concerns of rejection with switching to alternative agents. The sensory and motor symptoms recurred eight weeks after his initial IVIg treatment. He has since been maintained on IVIg, with good treatment response, although remission has not been achieved.

Patient 5

A 59-year-old woman with a history of end-stage renal failure of unknown aetiology received a left-sided kidney transplant 17 years before, followed by a right-sided transplant three years prior due to graft failure. She also had persistent BK viraemia. She was treated with tacrolimus for some months after the first transplant, which was switched to sirolimus but this was not tolerated due to hepatic dysfunction and dyslipidaemia, so was subsequently switched back to tacrolimus and she remained on this since, including after the second transplant. At the time of presentation, she was taking 2.5 mg of tacrolimus daily. She presented with a two-year history of progressive sensory disturbance in her feet, which spread to the proximal lower limbs and hands. For the last year, she had been developing increasing weakness, requiring crutches, eventually progressing to the point of causing multiple falls, which triggered admission to hospital. On initial assessment, she had profound lower limb weakness of hip flexors, ankle dorsiflexors and plantar flexors, as well as milder hand weakness. There was no sensory loss, but some hyperpathia in the lower limbs. Initial nerve conduction studies did not meet criteria for CIDP, with largely absent motor and sensory responses reported as suggestive of an axonal neuropathy (data now shown), but CSF demonstrated a markedly elevated protein of 332 mg/dL. She was treated with 2g/kg IVIg with good effect on her power increasing from MRC 1/5 to 4/5 in the lower limbs. However, she was unable to transition to sirolimus and required ongoing 6-weekly IVIg infusions. Repeat electrophysiology five months after starting IVIg was significantly improved, with emerging demyelinating features meeting the 2021 criteria for CIDP.

Patient 6

A 53-year-old man had a combined kidney-pancreas transplant for diabetic renal failure. He was immunosuppressed with tacrolimus 2 mg and prednisolone 7.5 mg, having previously been on mofetil mycophenolate, which was subsequently stopped due to leukopenia. Treatment with tacrolimus was started immediately after the transplant and two years later he developed a sensorimotor neuropathy. This initially involved his hands with weakness, tremor and loss of dexterity, then his lower limbs, and he progressed from being independently mobile to wheelchair-bound over a period of one month. The sensory disturbance was length-dependent and affected both legs and arms symmetrically. When first examined, he had proximal and distal weakness of lower and upper limbs, generalized areflexia, and loss of proprioception up to ankles and wrists. Serum protein electrophoresis with immunofixation revealed an IgG lambda paraprotein, CSF was acellular with normal protein (0.34 mg/dL), and electrophysiology demonstrated a sensorimotor demyelinating neuropathy with conduction slowing and block. MRI of the spinal cord was unremarkable. A diagnosis of tacrolimus-related CIDP in the context of longstanding diabetic neuropathy was made. Tacrolimus was stopped and replaced with azathioprine, and he was treated with three five-day courses of IVIg (each of 2 g/kg) six weeks apart. He made steady progress over the course of three months, ultimately recovering the capacity to stand independently and mobilize with the aid of a single stick.
