# Supplementary material for: Chronic Inflammatory Demyelinating Polyradiculoneuropathy (CIDP) Developing During Tacrolimus Treatment: A Case Series
Source: Muscle Nerve. 2025 Sep 17;72(6):1289–93. doi: 10.1002/mus.70025 (PMC12599595; doi:10.1002/mus.70025)
Supplement: Supplementary file 2 — Table S1: Supporting Information. [file MUS-72-1289-s001.docx]

Supplementary Table: summary clinical findings from previously reported patients with tacrolimus-related neuropathy.

| **Patient**  **(age [yrs], sex)** | **Daily tac. Dose (duration)** | **Transplant** | **Co-morbidities** | **Presentation** | **Other Ix** | **IVIg response?** | **Tacrolimus switched?** | **Long-term remission?** | **CIDP criteria met?** |
| --- | --- | --- | --- | --- | --- | --- | --- | --- | --- |
| Wilson et al. (1994)^10^  Patient 1  (57, M) | Dose n.s.  (2 weeks) | Liver | Type 2 diabetes  Hepatitis C | 6 months progressive  sensory loss 4 limbs  Prox. + dist. LL  weakness, hand weakness,  left foot drop | SPEP normal,  negative CMV/EBV  titres, ANA negative, CSF protein 0.89g/L | Not given  Response to  PLEX | n.s. | n.s. | 2021 |
| Wilson et al. (1994)^10^  Patient 2  (60, M) | 8mg  (2.5 mos) | Liver | Alpha-1 antitrypsin  deficiency | Prox. and dist.  weakness UL and LL, right foot drop, numbness UL and LLs. Progression >2 months | Deranged liver  function, normal ANA, CMV/EBV titres, ESR, SPEP, negative MAG Abs. CSF protein 1.12 g/L. | Yes  No response  PLEX | n.s. | n.s. | 2021 |
| Wilson et al. (1994)^10^  Patient 3  (35, M) | Dose n.s.  (2 weeks) | Liver | Alcohol-related  cirrhosis | Burning pain in feet, prox. + dist. LL weakness, mild UL weakness, length-depend.  sensory loss LL.  Duration n.s. | Deranged liver  function, normal SPEP, ANA, rheumatoid  factor, CSF protein  1.31 g/L | Yes | n.s. | n.s. | Neither 2010 nor 2021  Mild CV slowing, not meeting criteria |
| Labate et al. (2010)^11^  (56, F) | 5mg  (2 months) | Heart | Myocardial infarction  Heart failure | LL weakness worse on R, dist. only, normal UL  power, mild sensory loss  in feet. Duration n.s. | Normal B12/folate,  negative Hep B/C ,  CMV and HHV-6/7  serology, negative  ANA, paraneoplastic  antibodies, negative  ganglioside antibody, tacrolimus in range, CSF protein n.s. | n.s. | Yes – to cyclosporine | Yes | 2021 |
| Renard et al. (2012)^15^  (59, F) | Dose n.s.  (10 yrs) | Kidney | n.s. | Dysaesthesias hand and  feet, dist. sensory loss UL and LL, gait disorder, dysautonomia. Progressed  for ~3-4 months. | Raised tacrolimus  level (22.5 μg/L)  after onset of  symptoms, other  blood tests n.s.  CSF results n.s. | Yes | Yes – to sirolimus | Yes | 2021 |
| Echaniz-Laguna et  al. (2005)^14^  (62, M) | 10mg  (6 mos) | Liver | Hepatitis B and C | Dist. motor and sensory deficit in LL, hand weakness, progressing  over 6-8 weeks. | HIV-1/2 negative,  negative ganglioside  antibodies, CSF  protein 0.89 g/L,  nerve biopsy (sup.  peroneal)  demyelination and  segmental  demyelination | n.s. | No – dose increased | Yes | 2021 |
| Ayres et al. (1994)^17^  Patient 1  (58, M) | 0.3mg/kg  (8 days) | Liver | Primary sclerosing cholangiitis | Fluctuating flaccid  quadraparesis, facial weakness. Duration n.s. but not >2 months. | Magnesium 0.61  mmol/L, glucose  14.6 mmol/L, CSF  results n.s. | n.s. | Yes – to cyclosporine and azathioprine | Yes | Neither 2010 nor 2021  Axonal |
| Ayres et al. (1994)^17^  Patient 2  (31, M) | 0.3mg/kg  (8 days) | Liver | Fulminant hepatitis | Flaccid quadraparesis | Biochemistry screen normal, tacrolimus  3.4 ng/mL | n.s. | No | Not specified | Neither 2010 nor 2021  Axonal |
| Bhagavati et al.  (2007)^18^  Patient 1  (44, F) | 6mg  (10 mos) | Kidney | Focal segmental glomerulo-sclerosis | Facial + hand weakness,  Prox. + dist  LL weakness + sensory loss. Initial progression  <28 days, later relapse. | MAG and ganglioside  Abs negative,  negative Lyme, HIV &  hepatitis serology,  faint IgG-k band,  tarolimus 2.3-9.4  ng/mL, CSF protein  0.94 g/L | Yes | No | No  Relapse 28 months | 2021 |
| Bhagavati et al.  (2007)^18^  Patient 2  (53, M) | 24 then 14mg  (1 day) | Kidney | Hypertension | R hand finger numbness,  mild dist. LL weakness,  LL sensory loss.  Progression over days | Tacrolimus 7.3 ng/mL, other findings n.s. | n.s. | No – dose reduced | Yes | Neither 2010 nor 2021  Axonal |
| Boukriche et al.  (2001)^19^  (52, M) | Dose n.s.  (3 days) | Lung | Pulmonary fibrosis,  previous CMV | Flaccid areflexia, LL > UL. Burning  pain and numbness LL | Tacrolimus level  high >26 ng/mL,  creatinine 155,  normal magnesium  and glucose,  CSF protein normal | n.s. | No – dose reduced | Not specified | Neither 2010 nor 2021  Axonal |
| Bronster et al.  (1995)^20^  (45, M) | 10mg  (2 mos) | Liver | Cryptogenic cirrhosis | Prox. and dist.  weakness of UL and LL, dist. sensory loss.  Progression likely 2 months | Tacrolimus level  5.8 ng/mL, normal  electrolytes,  magnesium and  calcium | n.s. | Yes – to cyclosporine and azathioprine | Yes | 2021 |
| De Weerdt et al.  (1995)^21^  (45, M) | 0.04-0.08mg/kg  (9 days) | Liver and pancreas | Multiple endocrine  neoplasia 1 (MEN1),  panceatectomy,  type 3c diabetes,  parathyroidectomy,  thyroidectomy | Rapidly progressive  paraparesis, sensation normal, no UL  involvement. Timeframe  of progression n.s. but likely <28 days | Tacrolimus level 12 ng/mL, creatinine 1.8 mg/dL, urea 163 mg/dL, normal FBC and electrolytes  otherwise, normal  immunology and  infection screen. CSF protein 0.59g/L. | n.s. | No – dose reduction and added MMF | Yes | Neither 2010 nor 2021  Presentation in keeping with AMAN |
| Laham et al. (2001)^22^  (40, M) | 10mg  (2 weeks) | Kidney | n.s. | 4 limb weakness, dist. > prox. weakness,  Dist. sensory  loss. Progressed over 1- 2 weeks. | Tacrolimus level 22-30ng/mL at symptom onset, normal heavy metal screen, electrolytes, Vit B12 & folate, glucose. CSF n.s. | Yes | Yes – to cyclosporine | Yes | 2021 |
| Wu et al. (2013)^12^  (69, M) | 10mg  (7-20 days) | Kidney | TIA, coronary artery disease, MGUS, hypertension | Confusion and bilateral foot drop noted on d20 assesssment after transplant. ULs normal. Timeframe of progression n.s. but likely <28 days before nadir. | Tacrolimus level 48.1 ng/mL, MRI brain small vessel disease, normal electrolytes, heavy metal screen, thyroid function, vit B12 & folate. CMV/EBV/lyme serology negative. CSF protein normal | No | Yes – to cyclosporine | Yes | Bilateral common peroneal demyelination only |

Key: AMAN, acute axonal motor neuropathy; CKD, chronic kidney disease; CMV, cytomegalovirus; COPD, chronic obstructive pulmonary disease; CSF, cerebrospinal fluid; EBV, Epstein-Barr virus; ESR, erythrocyte sedimentation rate; FBC, full blood count; GN, glomerulonephritis; IF, immunofixation; IVIg, intravenous immunoglobulin; Ix, investigations; LL, lower limb; MAG, myelin associated glycoprotein; MGUS, monoclonal gammopathy of unknown significance; MRI, magnetic resonance imaging; n.s., not specified; RIJ, right internal jugular; SPEP, serum protein electrophoresis; Tac, tacrolimus; TIA, transient ischemic attack; Tx, treatment; UL, upper limb
